# Editorial: Infectious diseases (ID) - post-pandemic advances

**DOI:** 10.3389/fmicb.2023.1215690

**Published:** 2023-05-15

**Authors:** María de las Mercedes Pescaretti, Fabián E. Lopez, Mónica A. Delgado

**Affiliations:** ^1^Instituto Superior de Investigaciones Biológicas, CONICET-UNT, Instituto de Química Biológica “Dr. Bernabé Bloj”, Facultad de Bioquímica, Química y Farmacia, UNT, San Miguel de Tucumán, Argentina; ^2^Departmento of Natural Sciences School-Universidad Nacional de Chilecito, Chilecito, La Rioja, Argentina

**Keywords:** infectious diseases, post-pandemic COVID-19, bacterial, viruses, vaccines

Infectious diseases (ID) are disorders caused by microorganisms (bacteria, viruses, fungi, or parasites) living inside or outside of our bodies and are vital for the body functions. During colonization, important interactions between microorganisms and host take place. Depending on the host's immune response and on the microorganism‘s inoculum and genetic factors, some microorganisms can become pathogenic. These IDs can be transmitted directly (person-to-person contact) or indirectly (contact with contaminated surfaces, insect bites, or by contaminated food and water consumption). Taking in account the source and contamination levels, an ID can appear in a specific geographical area and time (epidemic); can be present permanently in a population (endemic diseases), or can be spread rapidly to several regions, countries and continents (pandemic). Some ID cause symptoms that can regularly be overcome, while other ID are life-threatening and may require hospitalization, increasing healthcare costs.

The objective of this topic was to create a space where scientific progress was reflected, determine the incidence and contribute to overcoming the problems produced by ID after the COVID-19 pandemic period, promoting the development of new rapid detection and control techniques that can be applied to improve human health. Approximately eighteen works were analyzed, seven of which contributed significant data to the pursued objective.

Xie et al. mentioned that the *Salmonella* Typhi and Paratyphi enteric fever continues to be a global public health problem. The incidence of Paratyphi serovar has increased, but only Typhi enteric fever vaccines have been developed and are available, but are ineffective to develop a crossed immune response against paratyphoid A fever. They propose that the development of a bivalent vaccine, effective for total eradication of paratyphoid A and other enteric fevers, is necessary and urgent.

Stilpeanu et al. collected data on human monkeypox, an emerging zoonotic disease caused by the monkeypox virus (mpox), which could spawn another global pandemic like the COVID-19 virus did. The increasing number of cases reported in areas beyond those endemic in Africa has led to its declaration as a global health emergency. However, the eradicated smallpox virus is similar to the mpox virus, both belong to the *Orthopoxvirus* viral genus, so vaccines and antivirals used against smallpox could serve to prevent mpox virus infection. They hold that taking into account the knowledge acquired during the COVID-19 pandemic, mpox virus infections and catastrophic effects on the population can be quickly avoided.

In the remaining four articles, written with original data, the COVID-19 virus was the study's subject. Gan et al. identified biomarkers associated with COVID-19's severity (age and physiological states of the patients). They analyzed nasopharyngeal, oropharyngeal, and anal samples, using transcriptomic techniques. Their data showed that oropharyngeal swabs showed higher microbial diversity. But in severe cases a negative correlation between COVID-19 and the microbiota‘s diversity was observed, probably due to antibiotic treatments. Wang et al. investigated the tuberculosis's effect related to COVID-19, mostly in China (the second country with highest tuberculosis incidence). They studied the clinical and epidemiological manifestations of COVID-19 (Omicron) and Tuberculosis (TB) (patients coinfected with the Omicron variant and TB and non-TB-COVID-19 patients), and reported that the Omicron variant is more contagious, but shows milder symptoms and a greater number of asymptomatic patients. The disease can rapidly progress to severe COVID-19, in patients with comorbidities or other chronic diseases, such as tuberculosis. Cao et al. reported that during the first months of the COVID-19 pandemic, rapid diagnosis of infected patients was essential, especially in asymptomatic patients or had mild symptoms of COVID-19. However, the asymptomatic people were not tested and they could infect other people. They developed the LAMP and RNA-LAMP technique for COVID-19 detection, and demonstrated that both have high specificity and sensitivity. They conclude that the LAMP technology will reduce diagnosis time and costs, apply diagnosis to the field, and increase the number of analyzed people. Goto et al. mentioned that in Japan, the people with two COVID-19 vaccine doses were susceptible to the Omicron variants. Therefore, they studied the neutralizing activity of 50% (NT50) for each variant, demonstrating that the Omicron's variants NT50s are the lowest. They also developed a model to predict the risk of viral infection, and showed that the ability to predict the Omicron BA1 and BA.2‘s NT50 was moderate; being the model of BA.1 that worked best in the data validation.

As a conclusion of the works published under this Research Topic, it is worth highlighting the opinion that Chong and Khan's mentioned in their article: “An isolated outbreak of a pathogen is capable of producing a pandemic that brings the world to a rapid halt for a reasonable period of time, crippling humanity with loss of life and battered health systems. This should be a reminder to keep scientific study active by designing effective interventions (vaccines, drugs, and diagnostics) and surveillance strategies, advancing effective progress on the catastrophic sacrifice that these agents produce”.

## Author contributions

All authors listed have made a substantial, direct, and intellectual contribution to the work and approved it for publication.

